# Genetic variation in *CDH13* gene was associated with non-small cell lung cancer (NSCLC): A population-based case-control study

**DOI:** 10.18632/oncotarget.22971

**Published:** 2017-12-05

**Authors:** Yingfu Li, Chuanyin Li, Qianli Ma, Yu Zhang, Yueting Yao, Shuyuan Liu, Xinwen Zhang, Chao Hong, Fang Tan, Li Shi, Yufeng Yao

**Affiliations:** ^1^ Department of Geriatrics, The No.1 Affiliated Hospital of Kunming Medical University, Kunming, 650032, China; ^2^ Institute of Medical Biology, Chinese Academy of Medical Sciences & Peking Union Medical College, Kunming, 650118, China; ^3^ Department of Thoracic Surgery, The No.3 Affiliated Hospital of Kunming Medical University, Kunming, 650118, China

**Keywords:** cadherin 13 (CDH13), single nucleotide polymorphisms (SNPs), non-small cell lung cancer (NSCLC), Chinese Han population

## Abstract

Cadherin 13 (CDH13, T-cadherin, H-cadherin) has been identified as an anti-oncogene in various cancers. Recent studies have reported that downregulation of H-cadherin in cancers is associated with *CDH13* promoter hypermethylation, which could be affected by the single nucleotide polymorphisms (SNPs) near CpG sites in the *CDH13* promoter. In the current study, we investigated and analyzed the association of seven SNPs (rs11646213, rs12596316, rs3865188, rs12444338, rs4783244, rs12051272 and rs7195409) with non-small cell lung cancer (NSCLC) using logistic regression analysis. SNPs rs11646213, rs12596316, rs3865188 and rs12444338 are located in the promoter region, rs4783244 and rs12051272 are located in intron 1, and rs7195409 is located in intron 7. A total of 454 patients with NSCLC were placed into a NSCLC group and 444 healthy controls were placed into a control group, all participants were recruited to genotype the SNPs using Taqman assay. Our results showed that the allelic frequencies of rs11646213 were significantly different between NSCLC and control groups (*P =* 0.006). In addition, the association analysis of these SNPs stratified into NSCLC pathologic stages I+II and III+IV showed that the allelic frequencies rs7195409 had a significant difference between NSCLC pathologic stages I+II and III+IV (*P =* 0.006). Our results indicated that the rs11646213 and rs7195409 in *CDH13* could be associated with NSCLC or its pathologic stages in the Chinese Han population.

## INTRODUCTION

Lung cancer (LC) is the leading cause of cancer deaths in the world. In 2012, it is estimated that there were more than 1.8 million new cases (13% of total cancer incidences) and almost 1.6 million deaths (20% of total cancer mortality) [1]. In China, the incidence and mortality of LC are reported to be approximately 0.7 and 0.6 million cases, respectively in 2013 [2]. Non-small cell lung cancer (NSCLC) accounts for approximately 80% of all LC cases [3]. Recently, several studies have shown that Cadherin 13 (CDH13, T-cadherin, H-cadherin) functioned as an anti-oncogene and that its polymorphisms were associated with the development of different cancers [4–8].

Cadherin 13, a new member of the cadherin superfamily, is coded by the *CDH13* gene, which maps to chromosome 16q24.2 [9]. Cadherin proteins often contribute to the formation of intercellular junctions (e.g. N- and E-cadherin). Loss of cadherin expression has been described in many epithelial cancers and may play a role in tumor cell invasion and metastasis [10]. Recent studies have reported that Cadherin 13 functioned as an anti-oncogene in lung [4], breast [5], ovarian [6], bladder [11], esophageal [12] and gastric [13]. As an anti-oncogene, the downregulation of Cadherin 13 expression would promote cancer progression. In 2001, Toyooka *et al.* reported that Cadherin 13 expression is diminished in LC, and they demonstrated that the downregulation of Cadherin 13 might be due to hypermethylation in the *CDH13* promoter [14]. In addition, Putku *et al.* described that single nucleotide polymorphisms (SNPs) in *CDH13* gene could affect the methylation of CpG sites in *CDH13* gene [15]. Moreover, studies have shown that the SNPs in *CDH13* gene could affect disease progression by influencing serum adiponectin levels [7,8], and the serum adiponectin level was identified to be associated with LC [16]. Thus, the SNPs in *CDH13* gene might be associated with LC through its correlation with *CDH13* gene methylation and serum adiponectin level. Several studies have reported that SNPs in *CDH13* gene were associated with other diseases, such as colorectal cancer [17–19]. However, few study investigated the association between SNPs in *CDH13* gene and NSCLC.

In the current study, we analyzed the association of seven SNPs (rs11646213, rs12596316, rs3865188, rs12444338, rs4783244, rs12051272 and rs7195409) in the *CDH13* gene with NSCLC and its pathologic stages in a Chinese Han population. SNPs rs11646213, rs12596316, rs3865188 and rs12444338 are located in the promoter, rs4783244 and rs12051272 are located in intron 1, and rs7195409 is located in intron 7.

## RESULTS

### Subject characteristics

Table 1 lists the clinical characteristics of the subjects in the present study. There were no significant differences in age or gender between the NSCLC and control groups (*P* > 0.05). In the NSCLC group, there were 283 patients with adenocarcinoma (AC), 163 patients with squamous cell carcinoma (SCC), and 8 patients with adenocarcinoma and squamous cell carcinoma (AC + SCC). There were 73 patients in pathological stage I, 73 patients in stage II, 163 patients in stage III and 145 patients in stage IV.

**Table 1:** Clinical characteristics of the subjects enrolled in the present study

	NSCLC	Control	*P*-value
N	454	444	
Age (years)	55.99 ± 10.76	54.82 ± 9.54	0.084
Sex (M/F)	307/147	296/148	
Adenocarcinoma (AC)	283		
Squamous cell carcinoma (SSC)	163		
Adenocarcinoma and Squamous cell carcinoma (AC+SSC)	8		
Clinical stage			
I	73		
II	73		
III	163		
IV	145		

### Association of the seven SNPs in *CDH13* with NSCLC

The allelic and genotypic frequencies for rs11646213, rs12596316, rs3865188, rs12444338, rs4783244, rs12051272 and rs7195409 in the NSCLC and control groups are listed in Table 2. These SNPs were all in Hardy-Weinberg equilibrium (HWE) for the NSCLC and control groups (*P* > 0.05). The logistic regression analysis showed that the allelic frequencies of rs11646213 were significantly different between NSCLC group and the control group (*P* = 0.006), which suggested that T allele of re11646213 had an increased effect on NSCLC risk after adjusted for gender and age (OR = 1.409;95%CI:1.105–1.798). However, the allelic and genotypic frequencies of the other SNPs were not significantly different between the NSCLC and control groups (*P* > 0.007).

**Table 2:** The association analysis between the seven SNPs in *CDH13* gene and NSCLC (After adjusted for gender and age)

SNPs	Genotypes (n,%)			*P*-value*	Alleles (n,%)		*P*-value*	OR (95%CI)
rs11646213	A/A	A/T	T/T		T	A		
NSCLC	292 (64.3%)	138 (30.4%)	24 (5.3%)	0.017	186 (20.5%)	722 (79.5%)	0.006	1.409 (1.105–1.798)
Control	316 (71.2%)	118 (26.6%)	10 (2.3%)		138 (15.5%)	750 (84.5%)		
rs12596316	A/A	A/G	G/G		G	A		
NSCLC	169 (37.2%)	217 (47.8%)	68 (15.0%)	0.131	353 (38.9%)	555 61.1%)	0.256	1.118 (0.923–1.354)
Control	192 (43.2%)	184 (41.4%)	68 (15.3%)		320 (36.0%)	568 (64.0%)		
rs3865188	A/A	A/T	T/T		T	A		
NSCLC	168 (37.0%)	218 (48.0%)	68 (15.0%)	0.242	354 (39.0%)	554 (61.0%)	0.311	1.104 (0.912–1.337)
Control	187 (42.1%)	190 (42.8%)	67 (15.1%)		324 (36.5%)	564 (63.5%)		
rs12444338	G/G	G/T	T/T		T	G		
NSCLC	178 (39.2%)	211 (46.5%)	65 (14.3%)	0.288	341 (37.6%)	567 (62.4%)	0.301	1.107 (0.913–1.343)
Control	196 (44.1%)	185 (41.7%)	63 (14.2%)		311 (35.0%)	577 (65.0%)		
rs4783244	G/G	G/T	T/T		T	G		
NSCLC	173 (38.1%)	212 (46.7%)	69 (15.2%)	0.536	350 (38.5%)	558 (61.5%)	0.442	1.078 (0.890–1.306)
Control	185 (41.7%)	193 (43.5%)	66 (14.9%)		325 (36.6%)	563 (63.4%)		
rs12051272	G/G	G/T	T/T		T	G		
NSCLC	189 (41.6%)	201 (44.3%)	64 (14.1%)	0.544	329 (36.2%)	579 (63.8%)	0.344	1.099 (0.904–1.334)
Control	201 (45.3%)	185 (41.7%)	58 (13.1%)		301 (33.9%)	587 (66.1%)		
rs7195409	A/A	A/G	G/G		G	A		
NSCLC	314 (69.3%)	129 (28.5%)	10 (2.2%)	0.436	149 (16.4%)	757 (83.6%)	0.176	1.195 (0.923–1.546)
Control	325 (73.2%)	110 (24.8%)	9 (2.0%)		128 (14.4%)	760 (85.6%)		

^*^Statistical significant threshold was set at *P* < 0.007 (0.05/7) determined by Bonferroni correction.

### Model of inheritance analysis of the seven SNPs in *CDH13* gene with NSCLC

Logistic regression analysis was used in model of inheritance analysis to evaluate the association between genotypes of the SNPs and NSCLC. The Akaike information criterion (AIC) and Bayesian information criterion (BIC) were calculated to determining the best fit inheritance model, which possesses the smallest AIC and BIC values. The best inheritance model with the lowest AIC and BIC for rs11646213 was the recessive model (*P* = 0.004, after adjusted for gender and age) (Table 3). In this model, the T/T genotype of rs11646213 conferred more risk of NSCLC (OR = 3.26; 95%CI:1.41–7.56). In addition, no significant differences for other SNPs were found between NSCLC and control groups in the model of inheritance analysis (*P* > 0.007) (data not shown).

**Table 3:** Different inheritance model analyses of rs11646213 in CDH13 between NSCLC and control groups (After adjusted for gender and age)

Models	Genotypes	Control (n, %)	NSCLC (n, %)	OR (95% CI)	*P*-value^*^	AIC	BIC
Codominant	A/A	316 (71.2%)	292 (64.3%)	1.00	0.011	1207.6	1490.8
	A/T	118 (26.6%)	138 (30.4%)	1.16 (0.84–1.61)			
	T/T	10 (2.2%)	24 (5.3%)	3.43 (1.47–8.00)			
Dominant	A/A	316 (71.2%)	292 (64.3%)	1.00	0.110	1212.0	1490.4
	A/T-T/T	128 (28.8%)	162 (35.7%)	1.30 (0.95–1.77)			
Recessive	A/A-A/T	434 (97.8%)	430 (94.7%)	1.00	0.004	1206.4	1484.8
	T/T	10 (2.2%)	24 (5.3%)	3.26 (1.41–7.56)			
Overdominant	A/A-T/T	326 (73.4%)	316 (69.6%)	1.00	0.640	1214.4	1492.8
	A/T	118 (26.6%)	138 (30.4%)	1.08 (0.78–1.49)			
Log-additive	---	---	---	1.38 (1.05–1.81)	0.019	1209.1	1487.5

^*^Statistical significant threshold was set at *P* < 0.007 (0.05/7) determined by Bonferroni correction.

### Linkage disequilibrium (LD) and haplotype analysis of the seven SNPs in *CDH13* gene

Significant LD values (*D’*> 0.85 and *R*^2^ > 0.7) among five of the seven SNPs (except for rs7195409 and rs11646213) were found in all individuals (Table 4). Based on the LD result, we constructed the haplotypes of the five SNPs (rs12596316, rs3865188, rs12444338, rs4783244 and rs12051272) and analyzed the difference in the haplotype frequencies (frequency more than 3%) between the NSCLC and control groups. Two main haplotypes were observed, and the frequencies of rs12596316A-rs3865188A-rs12444338G-rs4783244G-12051272G and rs12596316G-rs3865188T-rs12444338T-rs4783244T-12051272T were 58.0% and 33.7% in NSCLC and 59.8% and 30.6% in control groups. None of the haplotypes were significantly different in the NSCLC and control groups (*P* > 0.007) (Table 5).

**Table 4:** Linkage disequilibrium analysis of the seven SNPs in *CDH13* gene

D’ /R2 value	rs12596316	rs3865188	rs12444338	rs4783244	rs12051272	rs7195409
**rs11646213**	0.667/0.059	0.705/0.066	0.670/0.056	0.677/0.061	0.848/0.086	0.021/0.000
**rs12596316**	-	**0.937/0.867**	**0.914/0.795**	**0.867/0.749**	**0.918/0.760**	0.010/0.000
**rs3865188**	-	**-**	**0.967/0.879**	**0.910/0.822**	**0.963/0.826**	0.012/0.000
**rs12444338**	-	**-**	**-**	**0.972/0.895**	**0.967/0.886**	0.044/0.000
**rs4783244**	-	**-**	**-**	**-**	**0.979/0.860**	0.052/0.000
rs12051272	-	-	-	-	-	0.009/0.000

**Table 5:** Haplotype analysis of the five SNPs (rs12596316, rs3865188, rs12444338, rs4783244 and rs12051272) in *CDH13* gene

Haplotypes	NSCLC (n, %)	Control (n, %)	*P*-value^*^	OR(95%CI)
A A G G G^*^	526.47 (58.0%)	531.23 (59.8%)	0.221	0.881 (0.719∼1.079)
G T T T T^*^	306.14 (33.7%)	272.15 (30.6%)	0.221	1.135 (0.927∼1.390)

^*^Statistical significant threshold was set at *P* < 0.007 (0.05/7) determined by Bonferroni correction.

### Association analysis of seven SNPs in *CDH13* gene with different NSCLC pathologic stages

Table 6 lists the comparisons of the genotypic and the allelic distribution of the seven SNPs in different NSCLC pathologic stages (I+II and III+IV). The allelic frequencies of rs12444338, rs4783244, rs12051272 and the genotypic frequencies of rs7195409 just only exhibited a trend of significant difference between NSCLC I+II and III+IV patients (*P* = 0.011, 0.018, 0.024 and 0.013 respectively). However, the allelic frequencies of rs7195409 showed significant difference between NSCLC I+II and III+IV patients after Bonferroni correction (*P* = 0.006). We also conducted an inheritance model analysis to identify the best fit model of these four SNPs to compare the differences between NSCLC I+II and III+IV pathologic stages. The results showed that the best fit model for rs12444338, rs4783244 and rs12051272 was the log-additive model (*P* = 0.004, 0.006 and 0.005 respectively), and the best fit model for rs7195409 was the dominant model (*P* = 0.001) (Table 7).

**Table 6:** The association analysis between the seven SNPs in *CDH13* and NSCLC pathologic stages I+II and III+IV (After adjusted for gender and age)

SNPs	Genotypes (n, %)			*P*-value^*^	Alleles (n, %)		*P*-value^*^	OR(95%CI)
rs11646213	A/A	A/T	T/T		A	T		
III+IV	193 (62.7%)	98 (31.8%)	17 (5.5%)	0.578	484 (78.6%)	132 (21.4%)	0.313	1.199 (0.842–1.707)
I+II	99 (67.8%)	40 (27.4%)	7 (4.8%)		238 (81.5%)	54 (18.5%)		
rs12596316	A/A	A/G	G/G		A	G		
III+IV	108(35.1%)	151 (49.0%)	49 (15.9%)	0.358	367 (59.6%)	249 (40.4%)	0.137	1.247 (0.932–1.668)
I+II	61 (41.8%)	66 (45.2%)	19 (13.0%)		188 (64.4%)	104 (35.6%)		
rs3865188	A/A	A/T	T/T		A	T		
III+IV	106 (34.4%)	151 (49.0%)	51 (16.6%)	0.171	363 (58.9%)	253 (41.1%)	0.050	1.339 (1.001–1.792)
I+II	62 (42.5%)	67 (45.9%)	17 (11.6%)		191 (65.4%)	101 (34.6%)		
rs12444338	G/G	G/T	T/T		G	T		
III+IV	110(35.7%)	148 (48.1%)	50 (16.2%)	0.051	368 (59.7%)	248 (40.3%)	0.011	1.465 (1.090–1.969)
I+II	68 (46.6%)	63 (43.2%)	15 (10.3%)		199 (68.2%)	93 (31.8%)		
rs4783244	G/G	G/T	T/T		G	T		
III+IV	107 (34.7%)	149 (48.4%)	52 (16.9%)	0.074	363 (58.9%)	253 (41.1%)	0.018	1.426 (1.063–1.913)
I+II	66(45.2%)	63 (43.2%)	17 (11.6%)		195 (66.8%)	97 (33.2%)		
rs12051272	G/G	G/T	T/T		G	T		
III+IV	118 (38.3%)	142(46.1%)	48 (15.6%)	0.093	378 (61.4%)	238 (38.6%)	0.024	1.410 (1.047–1.899)
I+II	71 (48.6%)	59 (40.4%)	16 (11.0%)		201 (68.8%)	91 (31.2%)		
rs7195409	A/A	A/G	G/G		A	G		
III+IV	227 (73.7%)	75 (24.4%)	6 (1.9%)	0.013	529 (85.9%)	87 (14.1%)	0.006	0.599(0.416–0.861)
I+II	87 (60.0%)	54 (37.2%)	4 (2.8%)		228 (78.6%)	62 (21.4%)		

^*^Statistical significant threshold was set at *P* < 0.007 (0.05/7) determined by Bonferroni correction

**Table 7:** Different inheritance models analysis of rs12444338, rs4783244, rs12051272 and rs7195409 in *CDH13* gene between I+II and III+IV pathologic stages (After adjusted for gender and age)

SNPs	Models	Genotypes	I+II (n, %)	III+IV (n, %)	OR (95% CI)	*P*-value^*^	AIC	BIC
rs12444338	Codominant	G/G	68 (46.6%)	110 (35.7%)	1.00	0.012	600.8	831.4
		G/T	63 (43.1%)	148 (48.0%)	1.41 (0.87–2.30)			
		T/T	15 (10.3%)	50 (16.2%)	3.10 (1.41–6.82)			
	Dominant	G/G	68 (46.6%)	110 (35.7%)	1.00	0.031	603.0	829.5
		G/T-T/T	78 (53.4%)	198 (64.3%)	1.66 (1.05–2.65)			
	Recessive	G/G-G/T	131 (89.7%)	258 (83.8%)	1.00	0.009	600.7	827.2
		T/T	15 (10.3%)	50 (16.2%)	2.56 (1.22–5.38)			
	Overdominant	G/G-T/T	83 (56.9%)	160 (52.0%)	1.00	0.740	607.5	834.0
		G/T	63 (43.1%)	148 (48.0%)	1.08 (0.69–1.70)			
	Log-additive	---	---	---	1.64 (1.16–2.31)	0.004	599.5	826.0
rs4783244	Codominant	G/G	66 (45.2%)	107 (34.7%)	1.00	0.017	601.5	832.1
		T/G	63 (43.1%)	149 (48.4%)	1.40 (0.86–2.29)			
		T/T	17 (11.6%)	52 (16.9%)	2.91 (1.35–6.30)			
	Dominant	G/G	66 (45.2%)	107 (34.7%)	1.00	0.037	603.3	829.8
		T/G-T/T	80 (54.8%)	201 (65.3%)	1.64 (1.03–2.62)			
	Recessive	G/G-T/G	129 (88.4%)	256 (83.1%)	1.00	0.012	601.3	827.8
		T/T	17 (11.6%)	52 (16.9%)	2.41 (1.17–4.97)			
	Overdominant	G/G-T/T	83 (56.9%)	159 (51.6%)	1.00	0.760	607.5	834.0
		T/G	63 (43.1%)	149 (48.4%)	1.07 (0.68–1.69)			
	Log-additive	---	---	---	1.61 (1.14–2.27)	0.006	600.0	826.5
rs12051272	Codominant	G/G	71 (48.6%)	118 (38.3%)	1.00	0.015	601.2	831.9
		G/T	59 (40.4%)	142 (46.1%)	1.47 (0.91–2.38)			
		T/T	16 (11.0%)	48 (15.6%)	2.96 (1.34–6.51)			
	Dominant	G/G	71 (48.6%)	118 (38.3%)	1.00	0.024	602.5	829.0
		G/T-T/T	75 (51.4%)	190 (61.7%)	1.69 (1.07–2.69)			
	Recessive	G/G-G/T	130 (89.0%)	260 (84.4%)	1.00	0.015	601.7	828.2
		T/T	16 (11.0%)	48 (15.6%)	2.41 (1.14–5.08)			
	Overdominant	G/G-T/T	87 (59.6%)	166 (53.9%)	1.00	0.540	607.2	833.7
		G/T	59 (40.4%)	142 (46.1%)	1.15 (0.73–1.81)			
	Log-additive	---	---	---	1.63 (1.15–2.30)	0.005	599.6	826.1
rs7195409	Codominant	A/A	87 (60.0%)	227 (73.7%)	1.00	0.005	599.1	829.6
		A/G	54 (37.2%)	75 (24.4%)	0.46 (0.28–0.75)			
		G/G	4 (2.8%)	6 (2.0%)	0.37 (0.09–1.64)			
	Dominant	A/A	87 (60.0%)	227 (73.7%)	1.00	0.001	597.2	823.6
		A/G-G/G	58 (40.0%)	81 (26.3%)	0.45 (0.28–0.73)			
	Recessive	A/A-A/G	141 (97.2%)	302 (98.0%)	1.00	0.360	606.8	833.2
		G/G	4 (2.8%)	6 (2.0%)	0.50 (0.12–2.16)			
	Overdominant	A/A-G/G	91 (62.8%)	233 (75.7%)	1.00	0.003	598.7	825.1
		A/G	54 (37.2%)	75 (24.4%)	0.48 (0.29–0.78)			
	Log-additive	---	---	---	0.50 (0.32–0.77)	0.002	597.6	823.9

^*^Statistical significant threshold was set at *P* < 0.007 (0.05/7) determined by Bonferroni correction.

## DISCUSSION

Although persistent work has been done for the prevention and therapy of NSCLC, it remains the most common cancer in the world [20], and annual incidence and mortality has been trending upward [21]. Recently, studies have shown that polymorphisms in *CDH13* gene could lead to aberrant methylation of the *CDH13* promoter and influence plasma adiponectin level which have been demonstrated to be as biomarkers of LC [22–24]. However, they did not evaluate the association of SNPs in *CDH13* gene with LC [22–24]. In current study, we investigated the relationship between seven SNPs in *CDH13* gene and NSCLC.

Adiponectin is an adipose tissue-secreted protein that acts as an endogenous insulin sensitizer by binding to insulin receptors [25]. Data from recent studies proved that lower adiponectin levels are associated with an increased risk of endometrial cancer [26], renal cancer [27], colon cancer [28] and breast cancer [29]. In 2016, Wei *et al.* performed a meta-analysis on circulating adiponectin levels in various malignancies and found that decreased adiponectin levels are associated with the risk of various cancers, including LC [30]. Several studies revealed that SNPs (rs12444338, rs3865188, rs4783244, rs12051272, rs12596316, rs11646213, rs7195409) in *CDH13* gene determined plasma adiponectin levels in multi-ethnic populations [31–34]. In 2017, Nicolas *et al.* reported that rs11646213 was correlated with plasma adiponectin levels [34]. They observed that the A allele of rs11646213 was significantly associated with lower plasma adiponectin levels in Insulin Resistance Syndrome in French populations. However, this result was in contradiction with results of the present study, where our results showed that the T allele of rs11646213 might be the risk factor for NSCLC, which should be associated with lower plasma adiponectin levels. Moreover, Ling *et al.* found that rs7195409 was associated with adiponectin levels in Europeans (*P*  =  2.0 × 10^–5^) [35], while in Filipino women, Wu *et al.* [31] did not find any association between rs7195409 and adiponectin levels that was coincident with the current study (no association between this SNP and NSCLC). One of the reasons for the discrepancies might be the two different study populations (European and Asian) with different genetic backgrounds. The A allele of rs11646213 accounts for approximately 42% as the minor allele in the European population, while the same allele accounts for approximately 82% as the major allele in the East Asian population (http://asia.ensembl.org/Homo_sapiens/Variation/Population?db=core;r=16:82608546-82609546;v=rs11646213;vdb=variation;vf=6808482). Another reason could be that different diseases have different molecular mechanisms. The complex pathogenic mechanisms could make the same SNP play different roles in the different diseases. However, the plasma adiponectin levels were not measured in the current study, thus, we could not evaluate the association between genetic data, adiponectin levels and NSCLC risk. Whether rs11646213 was associated with NSCLC through influencing the plasma adiponectin levels in the current study population requires functional studies to be clarified in different diseases.

In 1996, Lee *et al.* found that the *CDH13* transcript was undetectable in all examined breast cancer and most other cancer cell lines, supporting its role as a tumor suppressor [36]. Then, Zhong *et al.* reported that the loss of Cadherin 13 expression was associated with tumorigenicity in nude mice transplanted with NSCLC tumors [37]. Moreover, Lee *et al.* and Chan *et al.* reported that the introduction and overexpression of Cadherin 13 in human breast carcinoma and hepatocellular carcinoma cell lines markedly inhibited tumor growth and invasiveness [38, 39]. Thus, Cadherin 13 was considered an important tumor suppressor in colorectal, lung, breast, ovarian and bladder cancers [40–43]. Aberrant methylation of tumor suppressor genes leads to tumorigenesis, due to the silencing of suppressors [44]. The aberrant methylation of CpG islands in the *CDH13* promoter might lead to aberrant gene expression and further promote tumor progression. This association was reported in many cancers, such as hepatocellular carcinoma, cervical neoplasia and breast cancer [45–47]. In 2016, Jin *et al.* reported that aberrant methylation of the *CDH13* promoter is associated with tumor progress in primary NSCLC [48]. Moreover, Shi *et al.* showed that genetic variability extensively influenced DNA methylation [49], and polymorphisms in the CpG sites of the *CDH13* promoter were associated with aberrant methylation of the *CDH13* promoter [15,50]. In the current study, our results showed that the frequency of rs11646213 in the *CDH13* promoter was significantly different between NSCLC and control groups at the allelic and genotypic level. Thus, we could deduce that rs11646213 might be associated with NSCLC through affecting the *CDH13* promoter methylation status. However, the other SNPs allelic frequency in the *CDH13* promoter, such as rs12596316, rs3865188 and rs12444338, was not significantly different between NSCLC and control groups. The reason for the discrepancy might be the linkage disequilibrium (LD) of these SNPs, and rs11646213 was not in LD with other SNPs in the *CDH13* promoter (Table 4).

On the NSCLC pathologic level, rs11646213 showed no association with NSCLC pathologic stages. It was interesting that rs12444338, rs4738244 and rs12051272 SNPs, which are in different regions of *CDH13* gene showed a correlation trend with NSCLC (*P* = 0.011, 0.018 and 0.024, respectively), which might be due to the fact that rs12444338 was in LD with the rs4783244 and rs12051272 (Table 4). In addition, Morisaki *et al.* reported that rs4783244 and rs12051272, which are located in *CDH13* intron 1, were in LD with rs12444338 located in the promoter because of a 30-kb haplotype block from the *CDH13* promoter region to the first intron [7]. Thus, rs4783244, rs12051272 and rs12444338 exhibited a similar trend of association with NSCLC pathologic stages, despite that these SNPs are located in different regions of the *CDH13* gene. In the current study, we found that rs7195409, located in *CDH13* intron 7, was associated with NSCLC pathologic stages (*P* = 0.006). The model of inheritance analysis showed that rs4783244, rs12051272, rs12444338 and rs7195409 were all associated with pathologic stages of NSCLC (*P* = 0.004, 0.006, 0.005 and 0.001, respectively). The best fit model of rs4783244, rs12051272 and rs12444338 was log-additive, but the best fit model of rs7195409 was dominant (Table 7). This difference could be because rs7195409 was not in LD with rs4783244, rs12051272 and rs12444338. The rs7195409 SNP was located in *CDH13* intron 7, and its surrounding nucleotide sequence does not match the known transcription factor binding site or miRNA targeted sequence. Thus, it is likely that rs7195409 is an independent marker or candidate SNP for NSCLC development. In addition, other SNPs surrounding or in LD with rs7195409 might be the real candidate SNPs that influence and play important roles in the development of NSCLC.

## MATERIALS AND METHODS

### Ethics statement

The current study was conducted in accordance with the guidelines and principles declared in the Declaration of Helsinki and approved by the Institutional Review Boards of the No.1 Affiliated Hospital of Kunming Medical University. All participants provided written informed consent.

### Subjects

A total of 454 patients (307 males and 147 females) diagnosed with NSCLC at the No.1 Affiliated Hospital of Kunming Medical University from July 2012 to May 2014 were recruited. The histological types were identified according to the World Health Organization (WHO 2004) classifications. The International System for Staging Lung Cancer was used to determine the lung cancer pathologic stages [51]. According to the pathomorphological reports, the NSCLC patients were divided into AC, SCC, and AC+SCC. Subjects with oncotherapy history or other cancers were excluded from the current study. In addition, individuals with hypertension, coronary heart disease and diabetes were also excluded from the current study. Clinical characteristics, such as gender, age, family history of cancer, and histological type of cancer, were collected. A total of 444 healthy individuals (296 males and 148 females) who had no family history of NSCLC were recruited from a population undergoing routine health checkups at the same hospitals. As subjects with a family history of cancer were excluded from control group, individuals who had family history of cancer were also removed from NSCLC group. All participants self-reported as Han and lived roughly within Yunnan Province, southwest of China.

### SNP genotyping using the TaqMan assay method

Genomic DNA of the subjects was extracted from peripheral lymphocytes using the QIAamp Blood Mini Kit (Qiagen, Hilden, Germany). Seven SNPs in the *CDH13* gene, namely, rs11646213, rs12596316, rs3865188, rs12444338, rs4783244, rs12051272 and rs7195409, were selected and genotyped through PCR amplification using a TaqMan assay. The TaqMan assays (primers and probes) were designed and produced by Applied Biosystems (Foster City, CA, USA). The assay ID for each SNP were rs11646213 (C__10076324_10), rs12596316 (C__31103274_10), rs3865188 (C__26380003_10), rs12444338 (C__26379996_10), rs4783244 (C__10076301_10), rs12051272 (C__31103269_10) and rs7195409 (C___1178915_10). The PCR reaction was performed in 384-well plates using a QuantStudio 6 Flex Fast Real-Time PCR system according to the manufacture instructions. A 5-μl reaction system consisting of 2.5 μl 2 × TaqMan Master Mix, 0.125 μl 40 × Primer and TaqMan Probe (FAM VIC) dye mix, 1.375 μl ddH_2_O, and 1 μl template DNA (substituted by equivalent ddH_2_O in negative control) was amplified using PCR cycle conditions: 95°C for 10 min; PCR stage 92 °C for 10 s and 60 °C for 1 min repeated in 40 cycles. Data acquisitions and analysis were performed on QuantStudio™ real-time PCR software. To identify the accuracy of SNP genotyping using the TaqMan assay, samples with each genotype of the seven SNPs were sequenced.

### Statistical analysis

Microsoft Excel software and the SPSS 19.0 statistical package (SPSS, Chicago, IL, USA) were used to perform statistical analyses. Both the NSCLC and control groups were evaluated by Hardy-Weinberg equilibrium (HWE) for representativeness. The linkage disequilibrium (LD) having a D’/R2 value greater than 0.85/0.70 was considered to be in linkage disequilibrium, and haplotypes were constructed based on the genotyping results by the expectation-maximization algorithm in SHEsis software [52, 53]. The effects of the polymorphisms on the risk of NSCLC were expressed as ORs with 95%CI, which were calculated using logistic regression analysis with adjustment for age and gender. The association between each genotype and the risk of NSCLC was assessed using inheritance model analysis of SNPstats software [54]. Five inheritance models were analyzed including Codominant, Dominant, Recessive, Overdominant and Log-additive (https://www.snpstats.net/snpstats/tutorial.htm?q=snpstats/tutorial.htm). Codominant model: this model allows every genotype to give a different and no additive risk., which compares heterozygous T/C (He) and homozygous for the variant allele C/C (Va) genotypes to the homozygous for the most frequent allele T/T. Dominant model: a single copy of C is enough to modify the risk, then heterozygous and homozygous genotypes have the same risk. Thus, we could compare a combination of these two possible genotypes T/C+C/C (Do) to the homozygous T/T. Recessive model: two copies of C are necessary to change the risk. Hence, T/C and T/T genotypes have the same effect. A combination of both T/T+T/C (Re) is compared to the variant allele homozygous genotype C/C. Over-dominant model: heterozygous are compared to a pool of both allele homozygous, the T/C (He) is compared versus T/T+C/C. Additive model: each copy of C modifies the risk in an additive form, the homozygous C/C have double risk than heterozygous T/C. Now, compare a combination of the two genotypes with weights 2 and 1 respectively 2C/C+T/C (Ad), to T/T. The AIC and BIC were calculated to determine the best fit model for each SNP. The statistical power was calculated using PS Software [55]. Bonferroni correction was performed on the *P* values for multiple comparison in the current study, and the statistical significant threshold was set at *P* < 0.007 (0.05/7).

## CONCLUSIONS

Several studies have demonstrated the association of SNPs in *CDH13* gene with the methylation of the *CDH13* gene and circulating adiponectin levels, which might be used as the diagnostic and prognostic biomarker for NSCLC [22]. In the current study, we found that the T allele of rs11646213 in *CDH13* might be the risk factor for NSCLC. The SNPs rs7195409 was associated with NSCLC pathologic stages. However, there were some limitations affecting the identification or association of SNPs with NSCLC in the current study. One of the limitations was the relatively modest sample size with a statistical power of only 75.9%. Another limitation was the lack of smoking status data for the control individuals, which made it difficult to perform further analyses of such exposure variables and to perform a gene-smoking interaction analysis in the current study. To clarify the association of *CDH13* variations with NSCLC susceptibility, larger scale samples and systemic studies that focus on the association of the SNPs in *CDH13* gene with the methylation status, serum adiponectin levels and NSCLC susceptibility are needed in the future
